# Minimizing Permanent Pacemaker Implantation After TAVR: Current Strategies, Monitoring Pathways, and Future Directions

**DOI:** 10.3390/jcm14217700

**Published:** 2025-10-30

**Authors:** Alfonso Reyes Mitre, Hector Lopez de la Garza, Claudio Espada Guerreiro, Dahyr Olivas Medina, Erick Marlon Avila Gil, Pablo Juan Salvadores, José Antonio Baz Alonso, Andres Iñiguez Romo, Victor Alfonso Jimenez Diaz

**Affiliations:** 1 Hemodynamic & Interventional Cardiology Unit, Cardiology Department, Hospital Alvaro Cunqueiro, University Hospital of Vigo, Estrada Clara Campoamor 341, 36312 Vigo, Spain; 2 Arrhythmia & Electrophysiology Unit, Cardiology Department, Hospital Alvaro Cunqueiro, University Hospital of Vigo, Estrada Clara Campoamor 341, 36312 Vigo, Spain; 3 Cardiology Department, Unidade Local de Saúde, Rua Conceição Fernandes, 4434-502 Vila Nova de Gaia, Portugal; 4 Cardiovascular Research Group, Galicia Sur Health Research Institute (IISGS) SERGAS-UVIGO, Estrada Clara Campoamor 341, 36312 Vigo, Spain

**Keywords:** transcatheter aortic valve implantation, permanent pacemaker implantation, right bundle branch block, left anterior fascicular block, cusp-overlap technique, electrophysiological study, membranous septum, balloon-expandable valve

## Abstract

Transcatheter aortic valve replacement (TAVR) has evolved over the last two decades into a cornerstone therapy for patients with severe symptomatic aortic stenosis. This therapy was initially reserved for those at high or prohibitive surgical risk but is now firmly established across all surgical risk categories. Its non-inferiority to surgical aortic valve replacement has been demonstrated even in low-risk populations, supporting the rapid worldwide expansion of its use. Nevertheless, despite procedural refinements and the advent of newer-generation prostheses, conduction disturbances leading to permanent pacemaker implantation (PPI) remain one of the most frequent and clinically relevant complications. Reported incidence ranges between 8% and 20% depending on prosthesis type, implantation technique, and baseline patient characteristics. Multiple clinical, anatomical, and procedural factors have been identified as strong predictors of post-TAVR conduction disturbances. Taken together, the integration of anatomical and clinical risk assessment, precise procedural planning, careful device selection, structured monitoring, and emerging therapeutic strategies constitutes a comprehensive, evidence-based approach to reduce the burden of conduction disturbances following TAVR. Such a multimodal framework has the potential not only to lower the incidence of permanent pacemaker implantation but also to improve safety, optimize healthcare resource utilization, and support the broader adoption of TAVR in increasingly younger and lower-risk patient populations.

## 1. Introduction

Over the last decade, transcatheter aortic valve replacement (TAVR) has solidified its role as a first-line treatment for patients with severe symptomatic aortic stenosis who are at moderate-to-high surgical risk, and more recently, its non-inferiority has been demonstrated in low-risk populations [1]. Since the first successful TAVR procedure in 2002, adoption has grown steadily, with over 1.5 million procedures performed worldwide in 2023 and annual volumes increasing across all risk categories [1].

Despite procedural refinements and newer-generation devices, conduction disturbances remain one of the most common and challenging complications following TAVR. Early data reported permanent pacemaker implantation (PPI) rates as high as 25–30% with first-generation valves, while current-generation devices have reduced this to 8–15%, depending on valve type and center experience [2]. These conduction disturbances—largely due to mechanical trauma from the prosthesis frame impinging on the conduction system—can result in a high-grade atrioventricular block, necessitating PPI.

Several predictive factors have been identified, including the length and morphology of the membranous septum [3], valve type, and implantation depth. Self-expanding valves are associated with higher PPI rates than balloon-expandable ones, largely due to their sustained radial force [4]. Balloon-expandable valves, by contrast, provide greater deployment precision and exert less delayed pressure on conduction tissue due to their lack of post-deployment expansion, making them a preferred choice in patients with pre-existing conduction disease.

**Figure A1:**
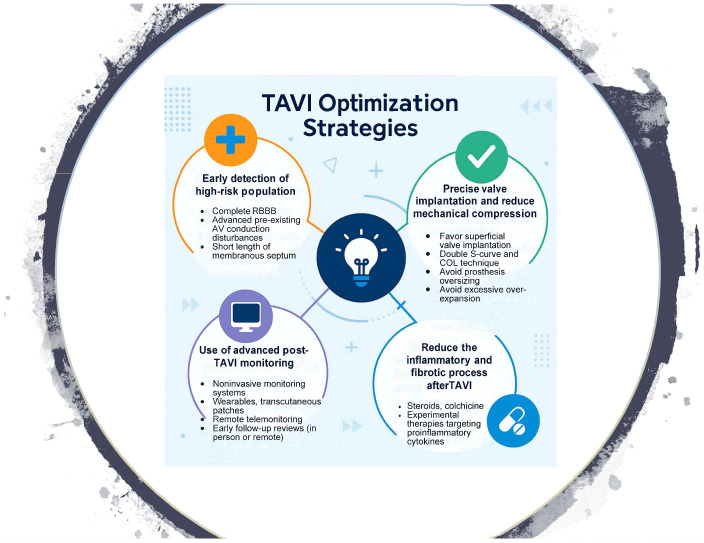
Central Image.

To mitigate these complications, an integrated strategy has emerged, ranging from the adaptation of valve implantation techniques to pharmacological interventions aimed at reducing post-TAVR edema and fibrosis, and structured monitoring systems that allow for early detection and timely treatment of conduction disturbances (Appendix A, Figure A1).

## 2. Procedural Techniques

- **The double S-curve and the cusp-overlap technique:** Recently introduced for self-expanding TAVR platforms, this method establishes the optimal fluoroscopic projection to guide valve implantation. Its objective is to achieve a shallower implantation at the aortic annulus, thereby reducing the risk of conduction disturbances by avoiding protrusion beyond the membranous septum, where the conduction system is more superficial. This strategy allows for more accurate control of implant depth and minimizes trauma to the conduction system, which may, in turn, decrease the incidence of permanent pacemaker implantation [5,6,7].
- **The membranous septum:** With current non-invasive imaging techniques, particularly computed tomography, the membranous septum can be accurately measured. Knowledge of its length provides valuable information for procedural planning, as it allows operators to perform higher valve implantations as a strategy to reduce the risk of high-grade atrioventricular block and, consequently, the need for permanent pacemaker implantation [8,9,10].

## 3. Patient and Valve Selection

Despite significant procedural and anatomical advances. Two key clinical factors remain strong predictors of the need for permanent pacemaker implantation (PPI) after TAVR. First, patients at intermediate or low surgical risk are increasingly undergoing TAVR, and observational data suggest that they may have a higher incidence of new PPI [11]. Second, the presence of underlying conduction abnormalities continues to be an independent risk factor for PPI [12].

- **Baseline Conduction Disorders and Valve Type Selection:** The presence of conduction abnormalities such as right bundle branch block (RBBB), left anterior fascicular block (LAFB), or bradyarrhythmias significantly increases the likelihood of permanent pacemaker implantation (PPI) after TAVR [11,12]. In the PARTNER trial, pre-existing conduction disturbances were independently associated with higher PPI rates, longer hospital stays, and a greater incidence of adverse clinical events [11]. In this context, balloon expandable valves are often preferred in patients with baseline conduction disease, as their greater implantation precision and reduced radial force on the conduction system are associated with lower PPI rates. Registry data suggest PPI rates as low as 4–8% with newer-generation balloon-expandable valves, compared with 10–20% for self-expanding valves [4], supporting their use as a strategy to mitigate conduction-related complications in higher-risk patients.

Despite advances in procedural techniques and patient selection, the inflammatory and fibrotic response triggered by prosthesis deployment remains a significant contributor to conduction system injury after TAVR. Therefore, attention has shifted toward strategies aimed at attenuating the inflammatory and fibrotic cascades responsible for post-TAVR conduction abnormalities.

## 4. Pharmacologic Strategies

Pharmacologic interventions aimed at modulating this inflammatory cascade have been proposed to reduce the incidence of conduction abnormalities and, consequently, the need for permanent pacemaker implantation. Although still in the exploratory phase, several therapeutic agents have been evaluated or are currently under investigation for this purpose.

- **Corticosteroids:** Corticosteroids were initially investigated for their potential to attenuate the post-TAVR inflammatory response and limit conduction tissue edema. Early reports were encouraging; however, subsequent larger studies failed to demonstrate a consistent reduction in PPI incidence, and concerns persist regarding systemic side effects and the lack of clear patient selection criteria [13,14,15].
- **Colchicine:** Colchicine, a well-established anti-inflammatory agent, is currently being evaluated in the Co-STAR trial (NCT04870424), a randomized, double-blind, placebo-controlled study. The trial aims to determine colchicine’s efficacy in preventing fibrosis-related conduction disorders and atrial arrhythmias after TAVR by dampening the inflammatory process. This trial is ongoing, with primary results expected in mid-2025. If successful, it could establish the first pharmacologic approach specifically designed to reduce post-procedural conduction disturbances.

## 5. Post-Procedural Monitoring

Monitoring after TAVR is essential for the prompt detection of conduction disturbances and to guide the need for permanent pacemaker implantation (PPI). Risk stratification based on early post-procedural ECG changes enables clinicians to differentiate between patients requiring immediate intervention and those who can be safely observed.

- **Electrocardiographic and Electrophysiological Surveillance:** Continuous ECG monitoring for up to 7 days is recommended by the ESC/EHRA guidelines, supported by evidence showing that more than 50% of high-grade atrioventricular block events occur within the first 72 h after TAVR, although a relevant proportion may still develop later during hospitalization. For this reason, ambulatory monitoring with external systems or implantable loop recorders may be extended up to 30 days in selected cases [16]. In addition, invasive electrophysiological study (EPS) may be considered from the third day after TAVR, particularly in patients with new-onset left bundle branch block (LBBB), PR interval > 240 ms, QRS duration > 150 ms, or marked prolongation (>20 ms) in those with pre-existing conduction disease. An HV interval ≥ 70 ms is widely regarded as predictive of high-grade atrioventricular (AV) block, although some studies have proposed alternative thresholds, such as HV ≥ 65 ms or a delta HV ≥ 13 ms when comparing pre- and post-procedural measurements [16,17]. Together, these strategies highlight the importance of combining extended non-invasive monitoring with targeted invasive evaluation to refine risk stratification and guide timely pacemaker implantation.

## 6. Telemonitoring and Early Discharge Programs

The integration of remote monitoring technologies has emerged as a valuable component in post-TAVR care, particularly as healthcare systems strive for safe early discharge strategies. Telemonitoring allows for real-time detection of delayed conduction disturbances while enhancing resource utilization and patient satisfaction.

- **TeleTAVI Study:** Demonstrated that early discharge supported by structured telemonitoring using artificial intelligence (AI) is both feasible and safe. The study included stratified discharge timing—very early (<24 h), early (24–48 h), and standard (>48 h)—coupled with daily follow-up through a virtual voice assistant using natural language processing. Patients in the early discharge arms had comparable 30-day event rates to those discharged later, while reporting high adherence and satisfaction with monitoring [18].
- Additionally, several studies are focused on pre-, intra-, and post-TAVR electrocardiographic monitoring to validate the performance of conduction disturbance risk scales (NCT05657912), estimate a reduction in disturbances through notifications during the procedure (NCT05465655), and monitor patients with pre-existing conduction disturbances or those that develop intra- or peri-procedurally after discharge [19,20].

## 7. Preventive Pacemaker Implantation

Although not routinely performed, preventive permanent pacemaker implantation (PPI) prior to TAVR has been proposed in selected high-risk patients as a strategy to avoid periprocedural complications and prolonged hospitalization. This approach is particularly considered in patients with baseline right bundle branch block (RBBB), a short membranous septum, or advanced conduction system disease, in whom the likelihood of developing high-grade atrioventricular block is significantly increased. In this subgroup, the risk of requiring urgent pacing support or emergent PPI is high, and anticipating this scenario with a planned implantation may improve safety and streamline management. Retrospective analyses from high-volume centers have reported that prophylactic PPI in such patients can shorten hospital stay and reduce the incidence of secondary complications associated with prolonged immobilization, including phlebitis, urinary tract infections, and delirium. Moreover, preventive PPI may simplify procedural logistics by reducing reliance on temporary pacing and minimizing the need for emergent pacemaker implantation, both of which are associated with greater procedural complexity, higher resource utilization, and potential hemodynamic compromise [21,22]. While this strategy remains controversial and is not currently endorsed by international guidelines, its role continues to be explored as part of a broader effort to optimize conduction management in high-risk patients undergoing TAVR.

## 8. Conclusions

Ongoing advancements in our understanding of clinical, anatomical, and procedural contributors to post-TAVR conduction disturbances have led to significant improvements in patient management. Predictive imaging, refined implantation techniques, and strategic valve selection now enable tailored interventions for patients at the highest risk. In parallel, innovations in ambulatory monitoring, artificial intelligence-based follow-up, and emerging anti-inflammatory therapies offer new opportunities for the timely detection and prevention of PPI. A multifaceted, evidence-based approach—incorporating these insights—has the potential not only to reduce PPI rates but also to enhance the safety, efficiency, and broader adoption of TAVR in increasingly younger and lower-risk populations.

## Data Availability

No new data were created or analyzed in this study.

## Abbreviations

The following abbreviations are used in this manuscript:

TAVR	Transcatheter Aortic Valve Implantation
PPI	Permanent Pacemaker Implantation
RBBB	Right Bundle Branch Block
LAFB	Left Anterior Fascicular Block
LBBB	Left Bundle Branch Block
EPS	Electrophysiological Study
